# Measurement precision matters: Accounting for uncertainty alters estimated memory trajectories across the cognitive spectrum

**DOI:** 10.1002/alz.71976

**Published:** 2026-11-04

**Authors:** Emily H. Trittschuh, Phoebe Scollard, Paul K. Crane, Seo‐Eun Choi, Jesse Mez, Andrew J. Saykin, Laura E. Gibbons, Shubhabrata Mukherjee

**Affiliations:** ^1^ Department of Psychiatry and Behavioral Sciences University of Washington Seattle Washington USA; ^2^ VA Puget Sound Health Care System, GRECC Seattle Washington USA; ^3^ Bordeaux Population Health Research Center University of Bordeaux, Inserm Bordeaux France; ^4^ Department of Medicine University of Washington Seattle Washington USA; ^5^ Department of Neurology Boston University Chobanian & Avedisian School of Medicine Boston Massachusetts USA; ^6^ Indiana Alzheimer's Disease Research Center and Center for Neuroimaging Indiana University School of Medicine Indianapolis Indiana USA; ^7^ Department of Medical and Molecular Genetics, Department of Radiology and Imaging Sciences Indiana University School of Medicine Indianapolis Indiana USA

**Keywords:** cognitive aging, longitudinal cognitive decline, measurement precision, memory impairment, precision‐aware modeling

## Abstract

**INTRODUCTION:**

Harmonized cognitive composites are widely used to study longitudinal cognitive decline. Although harmonization places scores on a common latent scale, measurement precision varies across visits, age, and diagnosis, but is typically ignored in longitudinal models.

**METHODS:**

We analyzed harmonized memory scores and time‐varying standard errors from the National Alzheimer's Coordinating Center Uniform Data Set. We compared a naïve mixed‐effects model that did not incorporate measurement uncertainty with two precision‐aware models: a frequentist precision‐weighted and a Bayesian measurement‐error model. Comparisons were based on estimated age of memory impairment.

**RESULTS:**

Accounting for measurement precision produced age‐dependent differences in estimated memory trajectories. Naïve models predicted higher memory scores at older ages in cognitively normal and mildly impaired participants but lower scores in Alzheimer's dementia (AD). Precision‐aware models estimated threshold crossing 1.5–3 years earlier than the naïve model.

**DISCUSSION:**

Incorporating visit‐specific measurement precision alters estimated cognitive trajectories and the timing of clinically interpretable memory impairment.

## Highlights

- Measurement precision varies across age and diagnostic groups, even for harmonized cognitive scores
- Incorporating measurement precision changes estimated memory trajectories in longitudinal models
- Precision‐aware models estimated clinically interpretable memory impairment approximately 1.5–3 years earlier than naïve models.
- Frequentist and Bayesian precision‐aware models supported the same overall conclusion, although some model‐specific differences were observed Accounting for measurement uncertainty may affect inference in cognitive aging studies and trials

## 1 BACKGROUND

Longitudinal studies of cognitive aging are central to understanding age‐related cognitive change, neurodegenerative disease progression, and the timing of clinically meaningful cognitive impairment. Over several decades, cohort studies have collected repeated cognitive assessments across broad age ranges and diagnostic stages, providing the empirical foundation for Alzheimer's dementia (AD) research.^1^, ^2^ These studies are necessarily heterogeneous in design, reflecting differences in the populations under study, cognitive assessment batteries, visit schedules, and primary scientific aims.^3^, ^4^

Latent variable methods based on confirmatory factor analysis (CFA) and item response theory (IRT) are widely used to estimate cognitive ability while accounting for differences in item characteristics and scaling, and provide estimates of measurement precision for the resulting latent scores.^5^, ^6^, ^7^, ^8^ One important application is the harmonization and co‐calibration of cognitive data across instruments, versions, and cohorts. These approaches have enabled large‐scale analyses of cognitive trajectories, genetic associations, and clinical trial outcomes that would otherwise be infeasible.

However, harmonization addresses differences in scale and content, not differences in measurement precision. A fundamental property of latent variable models is that measurement precision varies systematically across the latent trait continuum as a function of item information.^7^, ^9^, ^10^, ^11^, ^12^, ^13^ Measurement precision varies across the latent trait as a function of item information and is greatest in regions where the available items provide the most information. Depending on instrument design and item targeting, precision may be lower at one or both extremes of the scale. In the context of cognitive aging, measurement precision varies systematically across age, cognitive level, and diagnostic group, often declining among individuals with greater impairment due to floor effects, reduced item information, and increased variability.^7^

Despite widespread adoption of harmonized latent cognitive composites, most longitudinal analyses use models that do not incorporate observation‐specific estimates of measurement uncertainty.^14^, ^15^ This assumption is rarely stated explicitly. Under a conventional homoskedastic specification, observations are treated as equally precise conditional on the model, regardless of differences in their estimated measurement uncertainty. In randomized clinical trials estimating treatment effects, simplifying assumptions about measurement precision are often less problematic: randomization protects the treatment contrast from bias, and increased outcome noise primarily reduces statistical power.^7^ However, modern trial designs increasingly rely on longitudinal modeling, age‐based analyses, enrichment strategies using cognitive thresholds, and harmonized composite outcomes. In such settings, assumptions about measurement precision may influence estimated rates of decline and time‐to‐threshold analyses even when treatment assignment is randomized. These issues are particularly relevant for trials of disease‐modifying therapies in AD, where cognitive thresholds guide enrollment and harmonized composites serve as primary outcomes. In observational studies of cognitive aging, however, age is commonly used as the time scale, follow‐up is irregular, and measurement precision often varies systematically with both age and disease severity. Under these conditions, heterogeneity in measurement precision may influence estimated cognitive trajectories and their clinical interpretation if not explicitly accounted for.^7^, ^16^ In practice, this assumption treats observations with very different measurement uncertainty levels as equally informative, raising the possibility that trajectory estimates may depend on whether measurement precision is explicitly incorporated into the longitudinal model.

Here, we evaluate the consequences of omitting observation‐specific measurement uncertainty from longitudinal models of memory performance using data from the National Alzheimer's Coordinating Center (NACC) Uniform Data Set (UDS).^17^, ^18^ Building on prior work developing and evaluating harmonized cognitive domain scores in NACC, including their measurement precision properties,^19^ we provide the first systematic comparison of precision‐aware and conventional modeling approaches in longitudinal cognitive aging studies.

We compare a conventional mixed‐effects model with two precision‐aware alternatives: a frequentist model that down‐weights less precise observations,^20^, ^21^ and a Bayesian measurement‐error model that explicitly incorporates visit‐specific uncertainty into the likelihood.^22^, ^23^ Rather than focusing solely on statistical model fit, we emphasize the implications of these modeling choices for scientific inference by translating differences in estimated trajectories into clinically interpretable quantities, such as the estimated age at which memory performance crosses a threshold for clinically interpretable impairment. Our objective is to clarify how assumptions about measurement precision influence inferences about cognitive aging and disease progression, and to highlight implications for longitudinal studies and clinical trials that rely on harmonized cognitive outcomes.

**RESEARCH IN CONTEXT**

1. **Systematic review**: We reviewed prior literature on longitudinal cognitive aging, psychometric harmonization, and measurement precision. Previous studies have shown that item response theory‐based harmonization improves comparability of cognitive scores and that measurement precision varies across ability levels and disease stages. However, most longitudinal analyses do not incorporate observation‐specific measurement uncertainty, even when using harmonized cognitive outcomes.
2. **Interpretation**: This study demonstrates that ignoring visit‐specific measurement precision leads to age‐ and diagnosis‐dependent differences in estimated memory trajectories and delays the estimated timing of clinically interpretable impairment. These findings show that measurement precision is not merely a psychometric detail but a substantive modeling assumption that directly influences longitudinal inference.
3. **Future directions**: Future work should quantify uncertainty in clinically interpretable estimates, such as the estimated age at impairment, using precision‐aware approaches. Understanding how precision‐aware modeling affects downstream analyses, including genetic studies and clinical trial design, will help clarify its broader implications for studies that rely on longitudinal cognitive outcomes.

## 2 METHODS

### 2.1 Study sample

We analyzed longitudinal cognitive data from participants enrolled in studies contributing to the NACC UDS.^17^, ^18^ The analytic sample included 25,898 participants who had to have at least two cognitive assessments at distinct ages to support estimation of longitudinal change. Clinical diagnosis was defined using the NACC UDS diagnostic variable (*naccudsd*), and participants were classified as cognitively normal (NC; code = 1), mild cognitive impairment (MCI; code = 3), or AD (code = 4). Participants with other primary etiologies of cognitive impairment, including non‐AD dementias, were excluded.

### 2.2 Cognitive outcome and measurement precision

Memory performance was summarized using harmonized latent memory scores derived from CFA of neuropsychological test data collected in the NACC UDS. Harmonized memory scores were derived using a CFA model with the robust maximum likelihood (MLR) estimator in Mplus (version 8.2),^24^ as previously described,^19^ to enable longitudinal and cross‐study analyses. Briefly, the memory model included indicators of episodic memory and orientation, with secondary factors used to account for method‐related residual dependence when supported by the data, such as dependence between immediate and delayed recall from the same Logical Memory story. The item bank was placed on a common scale using anchor items with equivalent content and administration across studies. Item parameters were calibrated using the most recent visit from each participant, so that each person contributed one observation to the calibration model; the fixed item parameters were then used to generate factor scores and corresponding standard errors at every available visit. Because UDS 1/2 and UDS 3 used different cognitive batteries, item parameters for the two phases were estimated separately and linked to the existing item bank through common anchor items. Full indicator lists, factor structures, anchor‐item specifications, and scoring details are provided in Mukherjee et al.^19^ and its supplement.

Latent variable models provide both point estimates of an individual's cognitive performance and visit‐specific standard errors that quantify measurement uncertainty.^7^, ^10^, ^12^ Person‐ and visit‐specific standard errors correspond to the posterior standard deviation (SD) of the expected a posteriori (EAP) latent score estimates obtained from the CFA model. These are referred to here as time‐varying standard errors. These standard errors reflect the information available for each person and visit and may therefore vary with test availability, assessment protocol, response pattern, floor or ceiling effects, and model‐implied shrinkage. As demonstrated in prior NACC work, measurement precision for harmonized cognitive scores varies systematically across age, cognitive level, and diagnostic group, reflecting differences in item information across the latent ability range, including reduced precision at the extremes, as well as variation in test availability across visits.^19^ These time‐varying standard errors were incorporated into the present analyses to characterize observation‐level uncertainty in memory performance. Measurement precision was characterized across UDS waves (Figure 1a) and by clinical diagnosis (Figure 1b), based on the distribution of time‐varying standard errors.

**FIGURE 1:**
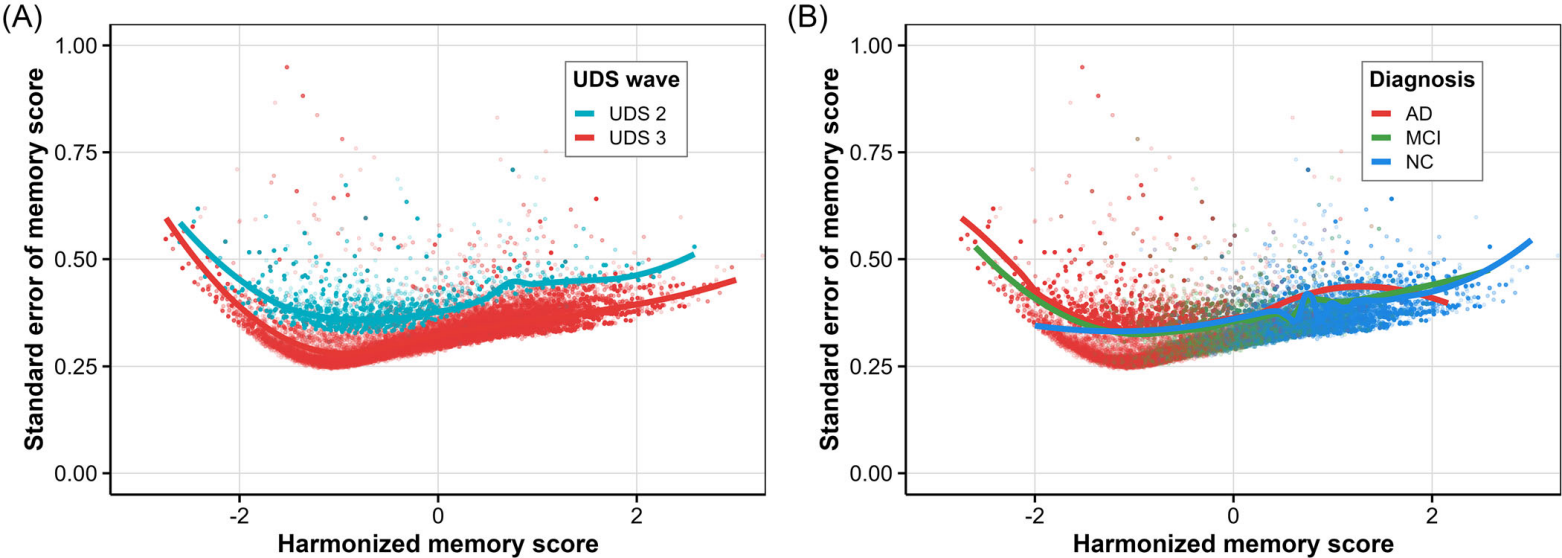
Measurement precision of the harmonized memory score. (A) UDS 3 memory scores show consistently lower SEs, that is, greater precision compared to UDS 1 and 2; (B) Measurement precision varies systematically across the memory scale and by clinical diagnosis, with increased uncertainty at floor and ceiling ranges, particularly in AD, and relatively lower SEs (higher precision) around 1 to 2, typical of NC and MCI individuals. AD, Alzheimer's dementia; MCI, mild cognitive impairment; NC, cognitively normal; SE, standard error; UDS, Uniform Data Set.

### 2.3 Statistical analysis

#### 2.3.1 Time scale and covariates

Age was used as the primary time scale and centered at age 60. All models were adjusted for sex, race, years of education, apolipoprotein E *(APOE)* ε4 carrier status, and baseline diagnostic group. Interaction terms between age and baseline diagnostic group were included to allow diagnosis‐specific rates of change. Missing data were handled using all available observations under the missing‐at‐random assumption, conditional on observed covariates, with likelihood‐based estimation in mixed‐effects models accounting for unbalanced data. Participant‐specific random intercepts and slopes were included to capture individual variability in baseline performance and age‐related change.

#### 2.3.2 Longitudinal modeling approaches

We compared three longitudinal modeling strategies. The frequentist precision‐weighted model and the Bayesian measurement‐error model below are referred to collectively as precision‐aware models.

- Naïve mixed‐effects model: A conventional linear mixed‐effects model with homoskedastic residual variance was fit without incorporating person‐ and visit‐specific CFA standard errors.^14^ Person‐ and visit‐specific standard errors were ignored, and all observations were treated as equally reliable measures of underlying memory performance. The model was fit using the *lmerTest* package (version 3.1‐3) in R (version 4.5.1).
- Frequentist precision‐weighted mixed‐effects model: A mixed‐effects model with an identical fixed‐ and random‐effects structure was fit, allowing residual variance to depend on visit‐specific measurement uncertainty. The frequentist model incorporated observation‐level weights of 1/(SE)^2^, where SE denotes the person‐ and visit‐specific standard error from the CFA model. This corresponds to inverse‐variance weighting, a standard approach that assigns greater weight to more precise observations (that is, those with smaller measurement variances).^16^, ^20^ This approach allows the residual variance to depend on measurement uncertainty while using standard maximum‐likelihood estimation. Models were fit using the *nlme* package (version 3.1) in R (version 4.5.1), with weights specified via the “weights” argument. This approach down‐weights observations with larger standard errors, thereby reducing the influence of less precise measurements on the estimation of population‐average trajectories and accounting for heteroscedastic measurement error in IRT‐based scores.^21^, ^25^
- Bayesian measurement‐error mixed‐effects model: A Bayesian mixed‐effects model, with the same fixed‐effects specification and correlated random intercepts and age‐specific random slopes as the frequentist model, treated observed memory scores as noisy realizations of an underlying latent cognitive trajectory, explicitly incorporating person‐ and visit‐specific standard errors into the likelihood. Specifically, observed scores were modeled as *y_ij_ ∼ N(µ_ij_, SE^2^_ij_ + σ^2^)*, where *y_i_*_j_ denotes the observed memory score, *µ_ij_* denotes the person‐specific true latent memory (including fixed and random effects) for person *i* at visit *j*, *SE_ij_* is the CFA‐derived standard error, and σ^2^ denotes the residual variance. The model included correlated random intercepts and age‐specific slopes to capture individual heterogeneity in baseline performance and rate of change. To ensure identifiability when combining measurement error correction with correlated random effects, we used informative priors on the variance components based on a preliminary uncorrelated model: *N(0.43, 0.01)* for the SD of random intercepts, *N(0.02, 0.001)* for the SD of random slopes, exponential(50) for the residual SD, and a weakly informative prior on the correlation between random intercepts and slopes which assigns higher probability to correlations near zero. Fixed effect priors were *N(0, 1)* for regression coefficients and *Student‐t(3, 0, 2.5)* for the intercept. The model was fit using the *brms* package (version 2.23) in R with the *cmdstanr* backend. Four Markov chain Monte Carlo (MCMC) chains of 3,000 iterations each (1,500 warmup iterations discarded) were run with tuning parameters adjusted to ensure robust sampling. Convergence was assessed using the Gelman‐Rubin statistic (R̂ < 1.01, indicating well‐mixed chains) and bulk effective sample sizes (ESS > 250, indicating adequate precision for parameter estimates).^22^, ^26^

#### 2.3.3 Population‐average predictions and model comparison

After fitting each model, we generated marginal (population‐average) predicted memory trajectories as a function of age, holding covariates at reference values. To facilitate numerical comparison across models, we obtained marginal population‐average predictions at ages 70, 80, 90, and 100 using the same covariate values for all models. Differences between naïve and precision‐aware estimated trajectories were evaluated across the age range and interpreted as discrepancies in predicted cognitive trajectories arising from the omission of measurement‐precision variation.

To quantify uncertainty in the estimated differences, we computed 95% confidence intervals (CIs) for the difference between the predicted trajectories of naïve and precision‐aware models. For frequentist models, CIs were derived via parametric bootstrap: we drew 1,000 samples from the asymptotic multivariate normal distribution of the fixed effects, computed predicted trajectories for each draw, and calculated pointwise 2.5th and 97.5th percentiles of the trajectory difference curves. For the Bayesian model, 95% credible intervals were computed directly from the posterior distribution by computing trajectory difference curves for 1,000 posterior draws of the fixed effects and taking pointwise quantiles.

To facilitate numerical comparison of predicted trajectories, we also summarized model‐based predictions at representative ages (70, 80, 90, and 100 years) for each diagnostic group. These values correspond to marginal predictions from each model and provide a concise summary of age‐dependent differences between naïve and precision‐aware approaches.

#### 2.3.4 Clinically interpretable threshold and time‐to‐threshold crossing

To facilitate interpretation on a clinically interpretable scale, we defined a threshold for memory impairment as 1 SD below the mean memory score for NC participants in our sample at age 60, a benchmark commonly used in studies of cognitive aging and impairment.^27^, ^28^ This threshold represents a clinically interpretable reference point rather than a validated clinical cutoff tied to functional outcomes. For each model, we estimated the age at which the population‐average predicted memory trajectory crossed this threshold, an approach analogous to change‐point estimation in longitudinal cognitive studies.^29^ Differences in estimated threshold‐crossing age between models were used to quantify how accounting for measurement precision influences clinically interpretable summaries of cognitive decline.

Uncertainty in threshold‐crossing age was quantified using 1,000 parameter draws. For the naïve and frequentist precision‐weighted models, fixed‐effect coefficients were drawn from their asymptotic multivariate normal distributions; for the Bayesian measurement‐error model, draws were sampled from the posterior distribution. For each draw, we generated the population‐average trajectory and identified the age at which it crossed the prespecified threshold by linear interpolation. The 2.5th and 97.5th percentiles of the resulting crossing‐age distributions were used to construct 95% CIs for the frequentist models and a 95% credible interval for the Bayesian model. Pairwise intervals were obtained from the distribution of differences between the corresponding model‐specific crossing ages.

## 3 RESULTS

### 3.1 Study sample

The analytic sample included participants from the NACC UDS with at least two longitudinal assessments of harmonized memory performance. Table 1 summarizes the sample's baseline demographic and clinical characteristics, overall and by diagnostic group (NC, MCI, and AD). As expected, participants with MCI and AD were, on average, older, had lower baseline memory performance, and were more likely to carry at least one *APOE* μ4 allele than NC participants.

**TABLE 1:** Baseline demographic and clinical characteristics of participants included in the analysis.

Characteristic	Overall	NC	MCI	AD
N	25898	12964	6307	6627
Age at baseline, years; mean (SD)	73 (7.9)	72.2 (7.8)	73.8 (7.6)	74.1 (8.3)
Female, %	57	65	48	49
Education, years, mean (SD)	15.6 (3.2)	16 (2.9)	15.5 (3.2)	14.8 (3.5)
*APOE* ε4 carrier, %	40	31	44	55
No. of visits, median (IQR)	4 (2–6)	5 (3–8)	3 (2–5)	3 (2–4)

Abbreviations: AD, Alzheimer's dementia; APOE, apolipoprotein E; IQR, interquartile range; MCI, mild cognitive impairment; NC, cognitively normal; SD, standard deviation.

Memory scores were available across a wide age range, with repeated observations per participant supporting estimation of age‐related change. Measurement precision of the harmonized memory score varied across both UDS waves and clinical diagnosis (Figure 1a,b). Figure 1a shows that UDS 3 assessments exhibit consistently lower standard errors than UDS 1 and 2, reflecting gains in measurement precision following test replacement. More importantly, Figure 1b demonstrates pronounced score‐dependent heteroskedasticity that differs by clinical diagnosis, with increased uncertainty toward the upper end of the score distribution in NC individuals and toward the lower end in AD,^30^ consistent with the observed distribution of measurement precision across the score range. These patterns violate the common assumption in naïve mixed‐effects modeling of homoskedastic residual variance, motivating the precision‐aware modeling approaches described below.

### 3.2 Model comparison

All three models estimated a moderate negative correlation between baseline memory and the rate of age‐related decline, ranging from −0.57 in the Bayesian model to −0.67 in the frequentist precision‐weighted model, indicating that baseline performance and subsequent change were related within the NACC sample (Table S1). Fixed‐effect estimates showed systematic differences in the handling of measurement precision: both the frequentist precision‐weighted and Bayesian measurement‐error models estimated slightly steeper age‐related decline ($\hat{\beta }$= −0.015) than the naïve model ($\hat{\beta \;}$= −0.013), with similar patterns for the age‐diagnosis interactions (Table S2). These parameter differences translated into age‐ and diagnosis‐dependent differences in predicted memory trajectories.

Figure 2 shows population‐average predicted memory trajectories by age for each modeling approach, stratified by baseline diagnostic group. Across groups, the naïve and the two precision‐aware models yielded similar predicted memory levels at younger ages. However, systematic differences emerged with advancing age, with both the magnitude and direction of differences varying by diagnostic group.

**FIGURE 2:**
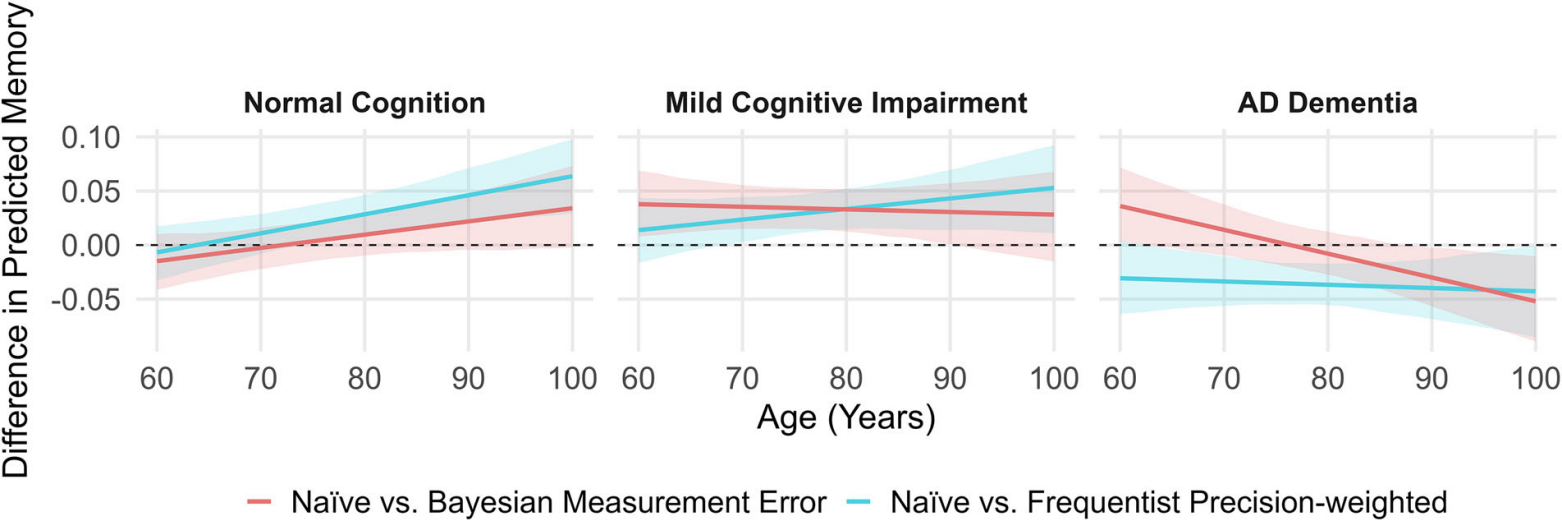
Age‐dependent differences in memory trajectories when measurement precision is ignored. Differences in predicted memory scores (Naïve−Precision‐aware) are shown as a function of age, stratified by baseline diagnosis. Solid lines represent differences relative to a frequentist precision‐weighted model (teal) and a Bayesian measurement‐error model (red). Positive values indicate higher predicted memory under the naïve model, whereas negative values indicate lower predicted memory. Shaded regions represent 95% confidence (frequentist) and credible (Bayesian) intervals. Differences increase with age and vary by diagnostic group, with the naïve model predicting higher memory at older ages in NC and MCI participants and lower memory in AD. AD, Alzheimer's dementia; MCI, mild cognitive impairment; NC, cognitively normal.

### 3.3 Age‐ and diagnosis‐dependent differences in predicted memory trajectories

Among NC and MCI participants, the naïve model predicted higher memory performance with increasing age compared with the two precision‐aware models. The naïve model also estimated later threshold crossing than the precision‐aware models. In contrast, among participants with AD, the naïve model predicted lower memory performance at comparable ages.

The frequentist precision‐weighted and Bayesian measurement‐error models produced generally similar trajectories in NC and MCI but differed more in AD at younger ages. Among participants with AD, the absolute difference between their predicted memory scores was 0.056 units at age 70, 0.038 at age 80, and 0.019 at age 90, with predictions converging by age 100. The pointwise 95% CIs and credible intervals for the naïve‐minus‐precision‐aware contrasts are shown as shaded regions in Figure 2. These intervals excluded zero at ages 75 and older in NC participants and across much of the observed age range in AD, whereas the pattern was less definitive in MCI, where the intervals were wider and largely included zero.

Table 2 summarizes predicted population‐average memory scores at representative ages across modeling approaches. The two difference columns represent the naïve model prediction minus the corresponding frequentist precision‐weighted or Bayesian measurement‐error prediction at each age. For the Naïve−Bayesian comparison, the naïve model predicted higher memory scores in NC and MCI across the selected ages. In AD, the naïve prediction was slightly higher at age 70, nearly identical at age 80, and lower at ages 90 and 100. Differences between naïve and precision‐aware models increase with advancing age and vary by diagnostic group. Among NC participants, the naïve model predicts progressively higher memory scores than precision‐aware models at older ages, with differences increasing from 0.011 units at age 70 to 0.064 units at age 100. Similar patterns are observed in participants with mild cognitive impairment. In contrast, among participants with AD, the naïve model predicts lower memory scores than precision‐aware models at comparable ages. Across diagnostic groups, the mean absolute difference between naïve and the precision‐aware predictions is approximately 0.04 units on the memory scale.

**TABLE 2:** Predicted population‐average memory scores at selected ages under alternative modeling assumptions

Diagnosis	Age (years)	Naïve	Frequentist precision‐weighted	Bayesian measurement error	Naïve−frequentist	Naïve−Bayesian
NC	70	0.912	0.901	0.906	0.011	0.006
	80	0.781	0.752	0.762	0.029	0.019
	90	0.650	0.603	0.619	0.046	0.031
	100	0.518	0.455	0.475	0.064	0.043
MCI	70	0.496	0.472	0.451	0.024	0.045
	80	0.319	0.286	0.277	0.033	0.042
	90	0.143	0.100	0.103	0.043	0.040
	100	−0.033	−0.086	−0.070	0.053	0.037
AD	70	−0.241	−0.208	−0.264	−0.034	0.023
	80	−0.454	−0.417	−0.455	−0.037	0.001
	90	−0.667	−0.627	−0.646	−0.040	−0.021
	100	−0.880	−0.837	−0.837	−0.043	−0.043

*Note*: Values are marginal population‐average predicted memory scores from each fitted longitudinal model, evaluated at the specified ages with covariates held at common reference values. The Naïve column contains predictions from the conventional mixed‐effects model that does not incorporate person‐ and visit‐specific measurement standard errors. The Naïve − Frequentist column is calculated as the naïve prediction minus the corresponding frequentist precision‐weighted prediction, and the Naïve − Bayesian column is calculated as the naïve prediction minus the corresponding Bayesian measurement‐error prediction. Positive values indicate higher predicted memory under the naïve model, whereas negative values indicate lower predicted memory.

Abbreviations: AD, Alzheimer's dementia; MCI, mild cognitive impairment; NC, cognitively normal.

Values correspond to marginal predictions from the longitudinal models described in the Methods section. Differences between naïve and precision‐aware models increase with age and vary by diagnostic group. Positive values indicate that the naïve model predicts higher memory scores than precision‐aware models, while negative values indicate lower predictions. The precision‐weighted and Bayesian estimates were generally similar in NC and MCI, whereas larger differences were observed in AD at younger ages and diminished with advancing age. Thus, incorporating measurement precision using either framework resulted in meaningful differences relative to the naïve model, although the specific choice of precision‐aware framework also influenced estimates in some portions of the age and diagnostic range.

### 3.4 Implications for estimated age at threshold crossing

To translate model differences into clinically interpretable quantities, we estimated the age at which population‐average predicted memory trajectories crossed a threshold of clinically interpretable impairment, defined as 1 SD below the mean memory score for NC participants at age 60, an a priori selected statistical benchmark.

The estimated population‐average trajectory crossed the prespecified impairment threshold at age 89.2 years under the naïve model (95% CI, 87.8–90.6), 86.2 years under the frequentist precision‐weighted model (95% CI, 85.2–87.3), and 87.7 years under the Bayesian measurement‐error model (95% credible interval, 86.6–89.0). Relative to the naïve model, threshold crossing occurred 3.00 years earlier under the frequentist precision‐weighted model (95% interval, 1.22–4.77) and 1.45 years earlier under the Bayesian measurement‐error model (95% interval, −0.32 to 3.24). The frequentist estimate was 1.54 years earlier than the Bayesian estimate (95% interval, −3.09 to 0.01). These differences reflect small but cumulative divergences in the predicted trajectories across age (Table 2).

## 4 DISCUSSION

In this study, assumptions about measurement precision meaningfully influence estimated trajectories of cognitive decline. Using harmonized memory scores from the NACC UDS, we compared a conventional mixed‐effects model that did not incorporate observation‐specific measurement uncertainty with two precision‐aware alternatives that incorporate visit‐specific uncertainty. Although all models produced similar predictions at younger ages, differences accumulated with advancing age and impairment, producing threshold‐crossing point estimates approximately 1.5–3 years earlier, though uncertainty differed across the two precision‐aware comparisons. These findings demonstrate that commonly used longitudinal models often omit available observation‐specific precision information, and relaxing this assumption can alter clinically interpretable summaries of cognitive trajectories.

Naïve models treat observations with differing measurement uncertainty as equally informative, which can alter estimated rates of decline. In these data, this led to higher predicted memory performance at older ages in NC and MCI participants and lower predicted memory in AD. Precision‐aware modeling modifies the assumed measurement model by incorporating observation‐level uncertainty. Since measurement precision tends to be lower toward the extremes of the score distribution, these observations contribute proportionally less to trajectory estimation. This reflects their reduced information content under the latent variable model, rather than a substantive decision to discount either the early or late stages of decline. Nevertheless, observations at the extremes may represent clinically important stages of disease, and lower statistical weight should not be interpreted as lower clinical importance. When reduced precision is concentrated at the upper or lower ends of the score distribution, down‐weighting these observations may alter the estimated trajectory. The alternative used in the naïve model treats observations as equally precise conditional on the model, without using the accompanying standard errors to distinguish their measurement uncertainty. Although the mean absolute difference between naïve and precision‐aware predictions was modest at approximately 0.04 units, this corresponded to roughly 3 years of average decline under the naïve model. Small slope differences can therefore accumulate with age and affect clinically interpretable summaries such as threshold‐crossing age.

These findings build on prior work showing that psychometric harmonization reveals heterogeneity in precision across ability levels and diagnostic groups^7^, ^19^ and extend methodological guidance on longitudinal cognitive modeling in aging populations.^31^ Complementary work by Nichols et al. showed that incorporating individual‐level measurement error in downstream analyses altered estimates of the proportion of cognitive variance explained by AD biomarkers, particularly for cognitive domains with greater measurement error.^32^ Our findings demonstrate that this heterogeneity has direct implications for longitudinal inference. The two precision‐aware models supported the same overall conclusion but were not interchangeable across the full age and diagnostic range.^7^, ^20^ Their modest differences may reflect their distinct treatment of uncertainty: inverse‐variance weighting changes the influence of individual observations, whereas the Bayesian model incorporates measurement variance directly into the likelihood. Trajectories were generally close in NC and MCI, with larger differences in AD at younger ages that diminished with advancing age. The frequentist precision‐weighted model estimated threshold crossing 3.00 years earlier than the naïve model, with a 95% interval excluding zero, whereas the Bayesian measurement‐error model estimated threshold crossing 1.45 years earlier than the naïve model, with an interval that included zero. The threshold‐crossing estimates from the two precision‐aware models did not clearly differ. Thus, both precision‐aware approaches differed from the naïve model, although the strength of evidence and some age‐ and diagnosis‐specific estimates varied.

The frequentist precision‐weighted model had a substantial computational advantage, requiring minutes compared to approximately 30 hours for the Bayesian model. Nevertheless, both precision‐aware models supported the same broad conclusion about differences between naïve and precision‐aware trajectories, suggesting that computational resources and the analyst's familiarity can guide method choice, while model‐specific differences may arise in some age and diagnostic ranges.

Harmonized latent scores are increasingly used in observational studies, genetic analyses, and clinical trials,^4^, ^19^, ^33^, ^34^, ^35^ but their accompanying precision information is often omitted from downstream models. Ignoring this information alters age‐specific predictions in ways that are difficult to detect without an explicit comparison. These patterns demonstrate heterogeneity in CFA‐derived measurement uncertainty that is not represented by the homoskedastic naïve model.^36^ Precision‐aware approaches that explicitly account for heterogeneity in measurement precision offer a principled means of addressing discrepancies in estimated cognitive trajectories and downstream analyses. These considerations may also extend to analyses of genetic, environmental, and clinical risk factors. When measurement precision varies systematically with age, disease severity, assessment composition, or the exposure itself, ignoring observation‐specific uncertainty could affect estimated associations and their standard errors. Evaluating the magnitude and direction of such effects across different downstream analyses is an important area for future work.

These findings also have implications for clinical trials, particularly when cognitive thresholds or longitudinal rates of decline are used. In such settings, assumptions about precision may influence trajectory estimates, time‐to‐threshold analyses, and sample size calculations.^10^

Similar issues may arise for commonly used endpoints such as the Clinical Dementia Rating–Sum of Boxes (CDR‐SB), Alzheimer's Disease Assessment Scale‐Cognitive Subscale (ADAS‐Cog), and Preclinical Alzheimer Cognitive Composite (PACC), which exhibit non‐uniform measurement properties across the impairment spectrum. Researchers often analyze these measures using models that do not account for observation‐specific differences in measurement precision. When precision varies systematically with cognitive level, ignoring this heterogeneity influences estimated trajectories and clinically interpretable summaries, whether the outcome is latent or a traditional total score.

These considerations extend beyond harmonized cognitive composites. Commonly used biomarkers in Alzheimer's disease research, including structural MRI, PET, and CSF measures, are typically analyzed as point estimates despite known measurement variability. Structural MRI measures are affected by scanner characteristics, head motion, and segmentation procedures^37^, ^38^, ^39^, while PET^40^, ^41^ and CSF measures are influenced by assay variability and preprocessing choices.^42^, ^43^ Yet downstream statistical analyses generally treat these quantities as exact observations. Depending on the modality and analytic pipeline, observation‐level uncertainty could be characterized using test–retest data, repeated image processing or segmentation, scanner‐ or assay‐specific variance components, or posterior or bootstrap distributions from quantitative measurement models. For example, uncertainty in MRI‐derived measures may reflect segmentation and processing variability, PET uncertainty may reflect image reconstruction and regional quantification, and CSF uncertainty may be informed by assay replication and laboratory quality‐control data. Such estimates would require modality‐specific validation before being incorporated into downstream analyses. Explicitly incorporating measurement uncertainty into statistical models, therefore, may represent a broader opportunity to improve inference across multiple modalities used to study cognitive aging and AD.

Precision‐aware modeling does not replace careful design or randomization, nor does it eliminate outcome variability. Instead, it more accurately represents the information content of observed data. By accounting for person‐ and visit‐specific uncertainty, these approaches may improve efficiency and sharpen inference, particularly when precision varies substantially across the outcome range.

Strengths include a large, well‐characterized dataset with repeated assessments across the cognitive spectrum. Our study addresses a practical setting commonly encountered in collaborative Alzheimer's research, where harmonized cognitive phenotypes and their associated standard errors are distributed for downstream analyses. We compared multiple modeling approaches to isolate the impact of incorporating measurement precision. We emphasized clinically interpretable quantities to connect statistical choices to substantive inference.

An alternative strategy would jointly estimate the measurement and longitudinal components within a full structural equation modeling framework, thereby propagating measurement uncertainty directly through the estimation process. Such approaches are statistically appealing when item‐level responses and calibrated measurement models are available. However, many collaborative Alzheimer's research initiatives and large‐scale psychometric harmonization efforts distribute harmonized cognitive phenotypes as latent scores with accompanying standard errors rather than item‐level data. In these settings, downstream analyses use the distributed scores, making methods that explicitly incorporate observation‐specific measurement uncertainty into longitudinal analyses both practical and broadly applicable. A joint measurement–longitudinal SEM could provide an informative benchmark, but with complex measurement models, irregular assessment timing, and many repeated visits, such models may be computationally difficult and require additional assumptions about the longitudinal structure.

Limitations should be noted. Since the true underlying cognitive trajectories are unknown, we cannot determine which modeling approach most accurately recovers them. Our analyses focused on population‐average trajectories rather than individual‐level prediction. We used an a priori statistical benchmark to define impairment; future work could evaluate thresholds tied to clinical outcomes such as changes in the global CDR. We did not propagate uncertainty in the standard errors themselves, which may slightly narrow intervals for the trajectory difference curves. Simulation and resampling studies are needed to quantify this effect and its implications for downstream analyses. We did not explicitly adjust for the UDS phase, as phase‐related differences in test composition are reflected in the harmonized scores and their visit‐specific measurement precision.

Harmonized cognitive outcomes are central to aging research, but commonly used longitudinal models do not incorporate the precision information accompanying these scores. Incorporating observation‐specific uncertainty produced systematically different trajectory estimates and threshold‐crossing summaries without changing the substantive scientific question. As harmonized composites are increasingly used in observational studies, genetic analyses, and clinical trials, precision‐aware modeling may improve the interpretation of cognitive decline and disease progression.

## CONFLICT OF INTEREST STATEMENT

Dr. Saykin acknowledges support from multiple NIH grants (P30 AG010133, P30 AG072976, R01 AG019771, R01 AG057739, U19 AG024904, R01 LM013463, R01 AG068193, R01 AG092591, T32 AG071444, U01 AG068057, U01 AG072177, U19 AG074879, as well as U24 AG074855). He has also received in‐kind support from Avid Radiopharmaceuticals, a subsidiary of Eli Lilly (PET tracer precursor) and Gates Ventures, LLC and Sanofi (Proteomics panel assays on IADRC and KBASE participants as part of the Global Neurodegeneration Proteomics Consortium), gift funds (GV) supporting technical contributions to the GRIP platform, funding to IU by the Alzheimer's Drug Discovery Foundation's Diagnostics Accelerator (ADDF), and he has participated in Scientific Advisory Boards (Bayer Oncology, Bristol Myers Squibb, Eisai, New Amsterdam, Novo Nordisk, and Siemens Medical Solutions USA, Inc) and an Observational Study Monitoring Board (MESA, NIH NHLBI), as well as External Advisory Committees for multiple NIA grants. He also serves as Editor‐in‐Chief of Brain Imaging and Behavior, a Springer‐Nature Journal. Author disclosures are available in the supporting information. The rest of the authors declare no conflicts of interest. Author disclosures are available in the Supporting Information.

## CONSENT STATEMENT

Ethical approval for this secondary analysis was reviewed by the University of Washington Institutional Review Board. Informed consent was not required as the study involved the secondary analysis of de‐identified data obtained from an approved National Alzheimer's Coordinating Center (NACC) data request.

## Supporting information




Supporting Information

**Supporting Information**:
