# Supplementary material for: Measurement precision matters: Accounting for uncertainty alters estimated memory trajectories across the cognitive spectrum
Source: Alzheimers Dement. 2026 Nov 4;22(11):e71976. doi: 10.1002/alz.71976 (PMC13736550; doi:10.1002/alz.71976)
Supplement: Supplementary file 2 [file ALZ-22-e71976-s001.docx]

**SUPPLEMENTAL TEXT**

**Supplementary Table S1. Random-effects variance components and the correlation between baseline performance and age-related decline across models.**

| **Component** | **Naïve** | **Frequentist Precision weighted** | **Bayesian Measurement error** |
| --- | --- | --- | --- |
| DSD(Intercept) | 0.63 | 0.62 | 0.54 (0.53, 0.55) |
| SD(age60) | 0.03 | 0.03 | 0.03 (0.03, 0.03) |
| Correlation | -0.65 | -0.67 | -0.57 (-0.59, -0.55) |
| Residual SD | 0.37 | 0.36 | ~0.00 (0.00, 0.01) |

**Note.** Correlation refers to the correlation between random intercepts and random slopes (age-related change). Values for the Bayesian model are posterior medians with 95% credible intervals. The near-zero residual variance in the Bayesian model reflects partitioning of observation-level variance into measurement error (SE²) and true residual variation (σ²), with the former dominating.

**Supplementary Table S2. Fixed effect estimates across naïve and precision-aware models.**

| **Parameter** | **Naïve** | **Frequentist precision-weighted** | **Bayesian Measurement error**  **(95% CrI)** |
| --- | --- | --- | --- |
| Intercept | 0.120 | 0.133 | 0.16 (0.12, 0.20) |
| Age (per year from 60) | -0.0131 | -0.0149 | -0.015 (-0.016, -0.014) |
| MCI (vs NC) | -0.372 | -0.392 | -0.42 (-0.45, -0.40) |
| Dementia (vs NC) | -1.072 | -1.048 | -1.12 (-1.15, -1.10) |
| Age × MCI | -0.0045 | -0.0037 | -0.003 (-0.004, -0.002) |
| Age × Dementia | -0.0082 | -0.0061 | -0.005 (-0.006, -0.004) |
| Female | 0.133 | 0.133 | 0.12 (0.11, 0.14) |
| Education (per year) | 0.049 | 0.049 | 0.05 (0.05, 0.05) |
| Race:  African-American  Other | -0.051  -0.079 | -0.048  -0.078 | -0.05 (-0.07, -0.04)  -0.08 (-0.11, -0.05) |
| *APOE* ε4 carrier | -0.247 | -0.248 | -0.23 (-0.24, -0.22) |

**Note.** All models adjusted for sex, years of education, race, and *APOE* ε4 carrier status. White was the reference category for race. NC was the reference category for diagnosis. CrI = credible interval.
